# Extracellular amylolytic activity in marine *Croceibacter* sp. 23-6: Cu^2+^ response and optimized wheat starch hydrolysis

**DOI:** 10.7717/peerj.21854

**Published:** 2026-11-03

**Authors:** Fangyan Jiang, Yuanyuan Zhang, Hongjin Zhang, Xiuqing Lin, Xiaoxiong Xu, Huimin Feng

**Affiliations:** 1 Tropical Biodiversity and Bioresource Utilization Laboratory, Qiongtai Normal University, Haikou, China; 2 Sanya Key Laboratory for Breeding of Tropical Crops, Hainan Tropical Ocean University, Sanya, China

**Keywords:** *Croceibacter* sp. 23-6, Extracellular amylolytic activity, Cu^2+^ response, Wheat starch hydrolysis, Response surface methodology, Mesopelagic marine bacterium

## Abstract

**Background:**

Marine bacteria are important sources of extracellular hydrolytic enzymes, but the amylolytic activity of many non-model marine bacterial lineages remains insufficiently characterized at the phenotype level. This study evaluated extracellular amylolytic activity in the marine isolate *Croceibacter* sp. 23-6 and examined its metal-ion response and wheat starch hydrolysis performance under defined laboratory conditions.

**Methods:**

Strain 23-6 was isolated from mesopelagic seawater of the western Pacific Ocean and identified at the genus level using 16S rRNA gene sequence analysis. Extracellular amylolytic activity was first screened on starch agar. A partially purified extracellular amylolytic protein preparation was then used to determine the effects of temperature, pH, thermal and pH stability, metal ions, apparent kinetic parameters and hydrolysis of different starch substrates. Wheat starch hydrolysis was further evaluated using single-factor experiments and response surface methodology based on a Box–Behnken design.

**Results:**

Strain 23-6 produced a clear starch-hydrolysis halo and was conservatively assigned to the genus *Croceibacter*. The partially purified extracellular protein fraction showed maximum amylolytic activity at 50 °C and pH 7.0 and retained comparatively higher activity under near-neutral conditions. Among the tested metal ions, Cu^2+^ increased the measured relative activity to 119.0% at 3 mM, whereas K^+^, Mn^2+^, Zn^2+^ and Fe^2+^ decreased the measured activity. Wheat starch showed the highest relative reducing-sugar release among the tested insoluble starch substrates. Response surface optimization predicted suitable hydrolysis conditions of approximately 52 min, 50 °C, 4.0 U/mL enzyme and 1.0% wheat starch. Validation under the adjusted conditions yielded 2.43 ± 0.09 mg/mL reducing sugars, expressed as maltose equivalents, consistent with the model prediction.

**Conclusions:**

These results provide strain-level evidence that *Croceibacter* sp. 23-6 exhibits extracellular amylolytic activity and indicate that its partially purified extracellular protein fraction displays a measurable Cu^2+^-responsive activity profile and wheat starch hydrolysis capacity. The findings define the functional properties of this marine bacterial extracellular amylolytic fraction while leaving the identity of the responsible enzyme(s), preparation homogeneity, and the mechanism of Cu^2+^ response for future work.

## Introduction

Culturable marine bacteria provide experimentally tractable systems for linking environmental isolates with extracellular hydrolytic phenotypes. Among these enzymes, α-amylases (EC 3.2.1.1) hydrolyse α-1,4-glycosidic linkages in starch and related α-glucans, generating maltooligosaccharides and reducing sugars relevant to starch conversion and microbial enzyme research (van der Maarel et al., 2002; de Souza & de Oliveira e Magalhães, 2010; Ashok et al., 2024; Ali et al., 2025). Marine-derived and extremophilic microorganisms have long been recognized as sources of hydrolases and other biocatalysts, including microbial amylases (Trincone, 2011; Dalmaso, Ferreira & Vermelho, 2015; Suriya et al., 2016). Recent studies continue to show that enzymes from culturable environmental microorganisms can be evaluated through enzyme production, purification, biochemical characterization and function-oriented assays, including microbial α-amylases and other extracellular hydrolases (Abo-Kamer et al., 2023; Devi et al., 2025; Adetiloye et al., 2025). However, most reported bacterial α-amylases still originate from well-studied taxa, particularly *Bacillus*, whereas phenotype-level evidence for extracellular amylolytic activity in many non-model marine bacterial lineages remains limited.

Marine polysaccharide degradation is a central microbial process in organic matter turnover and often depends on extracellular or cell-associated enzymes that determine substrate accessibility, product release and microbial interaction strategies (McKee et al., 2021; Thomas et al., 2021; D’Souza et al., 2023). Members of the family Flavobacteriaceae are particularly relevant in this context because they are frequently associated with the degradation and assimilation of high-molecular-weight polysaccharides in marine systems (Thomas et al., 2021; Yang et al., 2025). Nevertheless, ecological association or genome-based prediction alone does not demonstrate extracellular enzyme production by a given isolate. Direct phenotypic characterization of culturable strains is therefore needed to determine whether a specific isolate expresses measurable hydrolytic activity under defined experimental conditions.

*Croceibacter* represents a less frequently characterized marine lineage within the Flavobacteriaceae. The original description of *Croceibacter* atlanticus HTCC2559^T^ reported phenotypic degradation of starch and other macromolecules (Cho & Giovannoni, 2003), while its complete genome sequence subsequently provided further information on the metabolic potential of this genus (Oh et al., 2010). Quantitative biochemical characterization of extracellular amylolytic activity in Croceibacter isolates, however, remains limited. Accordingly, phenotypic and biochemical characterization of a culturable *Croceibacter* isolate can provide additional insight into strain-level extracellular amylolytic functions.

Beyond the detection of extracellular amylolytic activity, the metal-ion response of an enzyme preparation can provide a useful biochemical descriptor. Cu^2+^ is commonly reported to inhibit α-amylase activity, probably through interactions with catalytic or structural residues or through metal-induced conformational effects (Pasricha & Gahlot, 2022). Recent α-amylase studies further show that metal ions can markedly alter activity, stability and substrate hydrolysis profiles, including enzymes with strong activity toward wheat starch or other starch substrates (Ghevondyan et al., 2024; Radzlin et al., 2024). Therefore, documenting a Cu^2+^ response under controlled assay conditions can contribute to the functional characterization of an amylolytic preparation, although its mechanism requires future sequence- or structure-level validation.

Functional characterization should also be linked to a defined substrate-conversion context. Wheat starch provides a relevant α-glucan substrate for assessing whether extracellular amylolytic activity detected during strain screening can be translated into measurable reducing-sugar release. Response surface methodology, particularly Box–Behnken design, has been used in recent α-amylase studies to evaluate the combined effects of reaction variables and to optimize starch hydrolysis or enzyme production within defined experimental ranges (Abd-Elhalim et al., 2023; Adetiloye et al., 2025). Such optimization is best interpreted as laboratory-scale validation of hydrolysis performance under specified conditions, rather than as evidence of industrial readiness.

In this study, we isolated a starch-hydrolysing marine bacterium, designated strain 23-6, from mesopelagic seawater of the western Pacific Ocean and conservatively assigned it to the genus *Croceibacter* based on 16S rRNA gene analysis. We aimed to characterize the extracellular amylolytic phenotype of *Croceibacter* sp. 23-6 and evaluate the functional properties of its partially purified extracellular protein fraction. To this end, we examined temperature and pH profiles, stability, apparent kinetic parameters, metal-ion responses, hydrolysis of different starch substrates and wheat starch hydrolysis. We further used response surface methodology to evaluate wheat starch hydrolysis under defined reaction conditions. This work provides strain-level phenotypic and biochemical evidence for extracellular amylolytic activity in marine *Croceibacter* sp. 23-6, with particular attention to Cu^2+^ response and optimized wheat starch hydrolysis.

## Materials and Methods

### Sampling, isolation and screening

Seawater samples were collected from the mesopelagic layer of the western Pacific Ocean (130°04′42″E, 12°30′01″N) at a depth of approximately 300 m using a Niskin bottle. Approximately 1 L of seawater was aseptically collected, transported to the laboratory on ice, and processed within 24 h. For bacterial isolation, 1 mL of seawater was subjected to tenfold serial dilution (10^−1^–10^−6^) using sterile artificial seawater. The artificial seawater contained 24.0 g NaCl, 11.0 g MgCl_2_·6H_2_O, 4.0 g Na_2_SO_4_, 1.5 g CaCl_2_·2H_2_O, 0.7 g KCl and 0.2 g NaHCO_3_ per litre of distilled water.

Aliquots of appropriate dilutions (100 µL) were spread on starch agar (SA) plates. The screening medium was prepared with reference to media used for amylase-producing marine bacteria (Al-Agamy et al., 2021; Abirami et al., 2022), with minor adaptation for marine cultivation. The medium contained the following components per litre: 10.0 g soluble starch, 5.0 g peptone, 5.0 g yeast extract, 20.0 g NaCl, 0.5 g MgSO_4_·7H_2_O and 15.0 g agar. The medium was prepared using aged natural seawater, and the pH was adjusted to 7.5 before sterilization. Plates were incubated at 28 °C for 3–7 days. Morphologically distinct colonies were selected and repeatedly streaked on Marine Agar 2216 (BD Difco, Franklin Lakes, NJ, USA) to obtain pure cultures.

Primary screening for extracellular amylolytic activity was performed based on starch hydrolysis halos around colonies. Each purified isolate was spot-inoculated onto fresh SA plates and incubated at 28 °C for 48–72 h. Plates were then flooded with Lugol’s iodine solution containing 1% I2 and 2% KI. A clear halo surrounding the colony against the dark blue background was recorded as positive starch hydrolysis. Based on this qualitative screening, the isolate displaying the most pronounced halo formation under the screening conditions was selected for subsequent analysis and designated strain 23-6. During the experimental period, pure cultures were maintained at −80 °C in Marine Broth 2216 supplemented with 20% (v/v) glycerol.

### 16S rRNA gene sequencing and phylogenetic analysis

Genomic DNA was extracted from a fresh culture of strain 23-6 using a commercial bacterial DNA extraction kit (Tiangen Biotech, Beijing, China) according to the manufacturer’s instructions. The nearly full-length 16S rRNA gene was amplified by polymerase chain reaction (PCR) using the universal bacterial primers 27F and 1492R (DeLong, 1992). PCR amplification was performed in triplicate using a GeneAmp 9700 thermal cycler (Applied Biosystems, Foster City, CA, USA), with an annealing temperature of 58 °C, following a previously described procedure (Ye et al., 2023).

PCR products were examined by 1.0% (w/v) agarose gel electrophoresis. Amplicons of approximately 1.5 kb were excised, purified and submitted for bidirectional Sanger sequencing (BGI, Shenzhen, China). The resulting consensus sequence was compared with sequences in the NCBI GenBank and EzBioCloud databases for taxonomic identification (Yoon et al., 2017). The 16S rRNA gene sequence of strain 23-6 was deposited in GenBank under accession number PX796489.

For phylogenetic analysis, 16S rRNA gene sequences of closely related type strains were retrieved and aligned using ClustalW and MEGA 7.0 (Thompson et al., 1997; Kumar, Stecher & Tamura, 2016). A phylogenetic tree was constructed using the neighbour-joining method (Saitou & Nei, 1987). Evolutionary distances were calculated using the Kimura two-parameter model (Kimura, 1980), and the robustness of the tree topology was evaluated by bootstrap analysis with 1,000 replicates.

### Extracellular enzyme production and partial purification

For extracellular amylolytic protein production, strain 23-6 was first cultivated overnight in Marine Broth 2216 at 28 °C with shaking at 180 rpm. The seed culture was then inoculated at 1% (v/v) into the production medium. The production medium contained 20.0 g soluble starch, 10.0 g beef extract, 5.0 g yeast extract, 1.0 g K_2_HPO_4_ and 1.0 g NaCl per litre, prepared with aged natural seawater and adjusted to pH 7.5. Cultures were incubated at 28 °C and 180 rpm for 96 h.

After cultivation, the fermentation broth was centrifuged at 8,000× *g* for 20 min at 4 °C. The cell-free supernatant was collected as the crude extracellular enzyme extract. The crude extract was subjected to partial purification by ammonium sulfate fractionation, as described for microbial α-amylase purification with minor modifications (Abo-Kamer et al., 2023). Ammonium sulfate was first added to 40% saturation at 4 °C with continuous stirring. After standing for 2 h, the precipitate was removed by centrifugation at 12,000× *g* for 30 min at 4 °C. The supernatant was then brought to 70% saturation with additional ammonium sulfate and kept at 4 °C overnight. The precipitated proteins were collected by centrifugation, and the pellet was dissolved in a minimal volume of 20 mM Tris-HCl buffer (pH 8.0). The enzyme preparation was dialysed against the same buffer at 4 °C to remove residual salts and was subsequently used for biochemical characterization and hydrolysis assays. Because the preparation was obtained by ammonium sulfate fractionation and dialysis rather than purification to homogeneity, it is referred to throughout this study as a partially purified extracellular amylolytic protein preparation. Protein concentration was determined using the Bradford method with bovine serum albumin as the standard (Bradford, 1976).

### Amylolytic activity assay

Amylolytic activity was determined by measuring reducing sugars released from soluble starch using the 3,5-dinitrosalicylic acid (DNS) method (Miller, 1959). Unless otherwise specified, the standard reaction mixture contained 1.0 mL of partially purified extracellular amylolytic preparation and 1.0 mL of 1% (w/v) soluble starch prepared in 50 mM sodium phosphate buffer (pH 7.0). The stated 1% (w/v) refers to the substrate solution before mixing with the enzyme preparation, corresponding to a final starch concentration of 0.5% (w/v) in the 2.0-mL reaction mixture. Because total protein in the partially purified preparation does not specifically represent the amylolytic component, the standard activity assay used a fixed 1.0-mL aliquot rather than protein-based normalization. The enzyme solution was pre-incubated at 50 °C for 5 min, and the reaction was initiated by adding pre-warmed substrate. The reaction mixture was incubated at 50 °C for 10 min and terminated by adding 2.0 mL of DNS reagent. Colour development was achieved by boiling the mixture for 5 min.

After cooling to room temperature, distilled water was added to a final volume of 25 mL, and absorbance was measured at 540 nm using a spectrophotometer (Thermo Fisher Scientific, Waltham, MA, USA). Maltose standards ranging from 0.1 to 1.0 mg/mL were treated in the same manner to generate the calibration curve. One unit (U) of amylolytic activity was defined as the amount of enzyme preparation releasing 1 μmol of reducing sugar, expressed as maltose equivalent, per minute under the standard assay conditions. Reducing sugar yields in hydrolysis assays were also expressed as maltose equivalents. All activity values reported for the enzyme preparation therefore refer to reducing-sugar release under the specified DNS assay conditions.

### Temperature and pH effects on amylolytic activity

The effect of temperature on amylolytic activity was determined by measuring enzyme activity at 30 °C, 40 °C, 50 °C, 60 °C and 70 °C using the standard DNS assay. Each reaction was conducted for 10 min at the indicated temperature. The highest measured activity was defined as 100%, and relative activities were calculated accordingly.

The effect of pH on amylolytic activity was determined over the range of pH 4.0–9.0. The 1% (w/v) soluble starch substrate was prepared in 50 mM buffers: sodium acetate buffer for pH 4.0–5.0, sodium phosphate buffer for pH 6.0–7.0 and Tris-HCl buffer for pH 8.0–9.0. Reactions were performed at 50 °C, and the activity at the pH showing the highest activity was defined as 100%.

### Thermal and pH stability

Thermal stability was assessed by pre-incubating the enzyme preparation in 50 mM sodium phosphate buffer (pH 7.0) at 30 °C, 40 °C, 50 °C, 60 °C and 70 °C. Samples were withdrawn after 0.5, 1, 2, 4, 6 and 8 h, immediately cooled on ice, and residual activity was measured under the standard assay conditions. The activity before incubation was defined as 100%, and residual activities were calculated accordingly.

For pH stability, the enzyme preparation was pre-incubated at 50 °C in 50 mM buffers at different pH values: sodium acetate buffer at pH 5.0, sodium phosphate buffer at pH 6.0 and 7.0, and Tris-HCl buffer at pH 8.0 and 9.0. Samples were collected at the same time intervals, and residual activity was measured using the standard DNS assay. The initial activity under each pH condition was defined as 100%. Thus, this assay measured residual activity after exposure to the specified pH/buffer conditions at 50 °C.

### Effects of metal ions

The effects of metal ions on amylolytic activity were first evaluated at a final concentration of 3 mM using the standard DNS assay. CuSO_4_·5H_2_O, ZnSO_4_·7H_2_O, FeSO_4_·7H_2_O, MnSO_4_·H_2_O and CH_3_COOK were prepared as 0.1 M stock solutions and added to the reaction mixtures. These salts were used to evaluate the effects of Cu^2+^, Zn^2+^, Fe^2+^, Mn^2+^ and K^+^, respectively, on the activity of the enzyme preparation. Reactions without added ions served as controls. After incubation at 50 °C for 10 min, enzyme activity was measured and expressed relative to the control, which was defined as 100%.

Concentration-dependent assays were subsequently performed for all five ions. Final ion concentrations of 1, 2, 3, 4 and 5 mM were tested under the same assay conditions. Relative activities were calculated with reference to the ion-free control. These assays were used to characterize measured activity responses to the tested metal-ion conditions.

### Apparent kinetic analysis

Apparent kinetic parameters were determined using soluble starch as the substrate. Soluble starch concentrations of 0.5, 1, 2, 3, 5, 7, 9, 11 and 15.0 mg/mL were tested under the optimum assay conditions of 50 °C and pH 7.0. Initial reaction velocities were measured using the DNS assay. The replicate-level velocity data were fitted directly to the Michaelis–Menten equation by nonlinear regression, and apparent Km and Vmax values were estimated together with their 95% confidence intervals. Because the assays were performed with a partially purified extracellular enzyme preparation and soluble starch as a polymeric substrate, the kinetic values are reported as apparent parameters.

### Hydrolysis of different starch substrates and wheat starch hydrolysis assays

The hydrolysis profile of the partially purified extracellular amylolytic preparation was evaluated using six commercially available starch substrates purchased from local retail sources: corn starch, wheat starch, potato starch, cassava starch, pea starch and glutinous rice starch. All starch substrates were from commercially available food-grade products and were tested under the same preparation and reaction conditions. Soluble starch was included as the reference substrate and defined as 100%. The starches were used without prior gelatinization or other pretreatment. Each substrate was prepared as a 1% (w/v) suspension in 50 mM sodium phosphate buffer (pH 7.0), with reference to previously reported α-amylase assays using starch substrates (Chen et al., 2021; Albalawi et al., 2025).

For each assay, 1.0 mL of pre-warmed enzyme preparation was mixed with 1.0 mL of substrate suspension and incubated at 50 °C for 30 min. The substrate suspensions were thoroughly mixed before use. Corresponding substrate blanks were processed in parallel and used for background correction, and insoluble material was removed by centrifugation before absorbance measurement. The reaction was terminated with DNS reagent as described above, and reducing sugars were quantified as maltose equivalents. Relative reducing-sugar release was calculated by comparing reducing sugar production from each insoluble starch substrate with that from soluble starch. The assay was used to compare reducing-sugar release from different starch substrates under the same reaction conditions.

### Single-factor experiments for wheat starch hydrolysis

Because wheat starch showed the highest relative reducing-sugar release among the tested insoluble starch substrates, it was selected for hydrolysis optimization. Single-factor experiments were conducted to determine suitable ranges for hydrolysis time, temperature, enzyme dose and substrate concentration. In each experiment, one variable was changed while the remaining variables were kept constant. The reference conditions were 50 °C, 4 U/mL enzyme dose and 1.0% (w/v) wheat starch, with the reaction time adjusted according to the tested variable. The tested levels were 15, 30, 60, 80 and 120 min for hydrolysis time; 40 °C, 45 °C, 50 °C, 55 °C and 60 °C for temperature; 1, 2, 4, 6 and 8 U/mL for enzyme dose; and 0.1%, 0.5%, 1.0%, 1.5% and 2.0% (w/v) for wheat starch concentration. Reducing sugar yield, expressed as maltose equivalents, was used as the response variable. The single-factor results were used to define narrower experimental ranges for response surface optimization.

### Box–Behnken design and response surface optimization

A four-factor, three-level Box–Behnken design was used for response surface methodology (RSM) optimization based on the single-factor results. The use of RSM followed general principles for multivariable optimization and α-amylase-related process optimization studies (Bezerra et al., 2008; Bandal et al., 2021; Abd-Elhalim et al., 2023). The independent variables were hydrolysis time (A), temperature (B), enzyme dose (C) and wheat starch concentration (D). The coded and actual levels were 40, 60 and 80 min for hydrolysis time; 48 °C, 50 °C and 52 °C for temperature; 3, 4 and 5 U/mL for enzyme dose; and 0.8, 1.0 and 1.2% (w/v) for substrate concentration.

Design-Expert software (version 13.0.14; Stat-Ease Inc., Minneapolis, MN, USA) generated 29 randomized experimental runs, including five centre points. For each run, reducing-sugar yield was expressed as maltose equivalents. The data were fitted to a second-order polynomial model. Model significance and the significance of individual terms were evaluated by analysis of variance (ANOVA). Model adequacy was assessed using the coefficient of determination (R^2^), adjusted R^2^, predicted R^2^ and lack-of-fit test. Numerical optimization was performed to maximize reducing-sugar yield expressed as maltose equivalents. The four independent variables were constrained within their experimental ranges of 40–80 min for hydrolysis time, 48–52 °C for temperature, 3–5 U/mL for enzyme dose and 0.8–1.2% (w/v) for wheat starch concentration. The response was set to maximize, with lower and upper limits of 1.02 and 2.51 mg/mL, respectively; weights of 1 and an importance level of +++ were used for all criteria. Graphical optimization defined the feasible region using a minimum predicted reducing-sugar yield of 2.40 mg/mL. The predicted optimal conditions were adjusted to practical experimental values before validation. The validation experiment was performed under the adjusted conditions, and the experimentally obtained reducing-sugar yield was compared with the model-predicted response at the same conditions. The response surface model was interpreted only within the tested factor ranges.

### Statistical analysis

Unless otherwise stated, all quantitative assays were performed in triplicate. For quantitative enzyme and hydrolysis assays, triplicate measurements consisted of three independently prepared reaction mixtures, each of which underwent the complete enzymatic reaction and DNS measurement procedure. Data are presented as mean ± standard deviation. Statistical analyses were conducted using IBM SPSS Statistics 26.0 (IBM Corp., Armonk, NY, USA). One-way ANOVA followed by Tukey’s multiple comparison test was used for single-factor comparisons of endpoint activity measurements, with *p* < 0.05 considered statistically significant. Thermal- and pH-stability time courses were summarized descriptively as mean ± SD and were not subjected to the one-way ANOVA used for endpoint comparisons. For RSM optimization, ANOVA in Design-Expert was used to evaluate model significance and adequacy.

## Results

### Screening and genus-level identification of an extracellular amylolytic marine bacterium

A starch-hydrolysing bacterial isolate, designated strain 23-6, was selected from mesopelagic seawater of the western Pacific Ocean on the basis of its extracellular amylolytic phenotype. After iodine staining, strain 23-6 produced a distinct transparent halo around the colony on starch agar, indicating extracellular starch hydrolysis (Fig. 1). The nearly full-length 16S rRNA gene sequence of strain 23-6 was obtained and deposited in GenBank under accession number PX796489. BLASTn and EzBioCloud analyses placed the strain within the genus *Croceibacter*. Phylogenetic analysis showed that strain 23-6 clustered with *Croceibacter atlanticus* HTCC2559^T^, sharing 98.95% 16S rRNA gene sequence similarity (Fig. 2). Because species-level assignment cannot be resolved reliably by 16S rRNA gene similarity alone without genome-based taxonomic evidence, the isolate was conservatively designated *Croceibacter* sp. 23-6. Thus, strain 23-6 represents a culturable marine *Croceibacter* isolate with a confirmed extracellular starch-hydrolysing phenotype.

**Figure 1:**
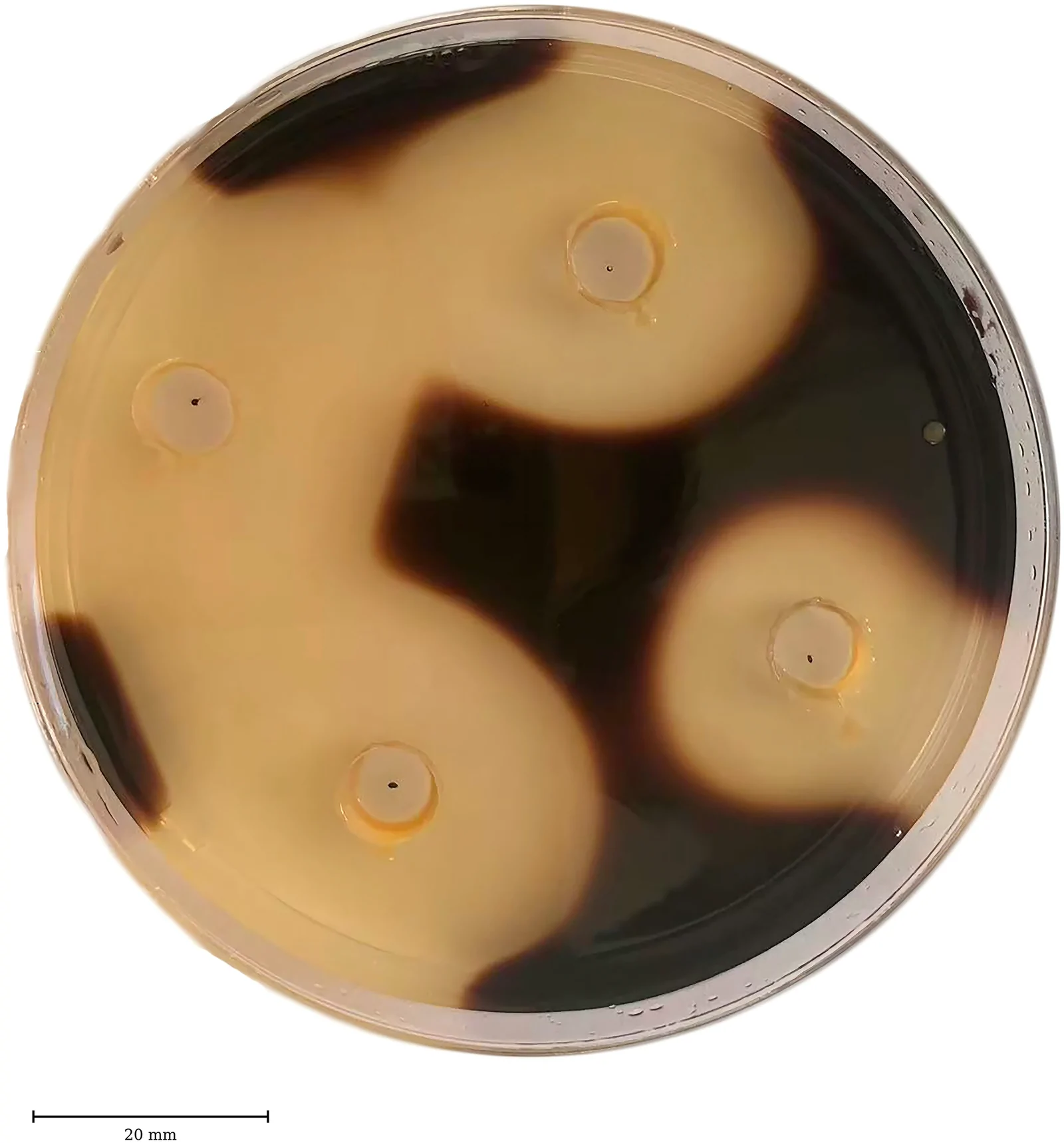
Starch hydrolysis zone of isolate strain 23-6. The formation of a clear halo on starch agar following iodine staining provides qualitative evidence of starch hydrolysis by strain 23-6. Scale bar = 20 mm.

**Figure 2:**
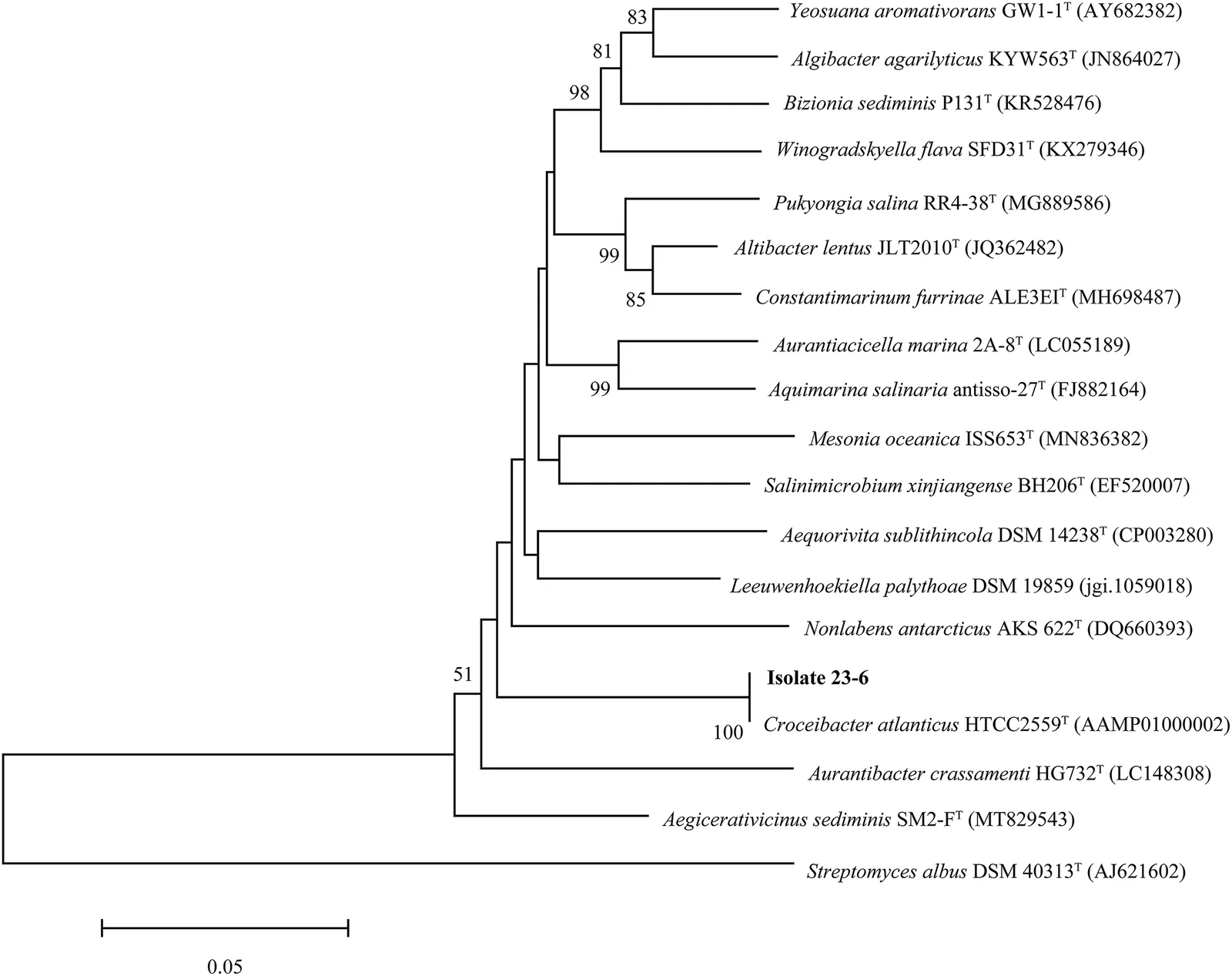
Phylogenetic analysis of strain 23-6. Neighbor-joining tree based on 16S rRNA gene sequences showing the phylogenetic position of strain 23-6. The strain forms a clade with *Croceibacter atlanticus* HTCC2559^T^, sharing 98.95% sequence similarity. Bootstrap values based on 1,000 replicates and greater than 50% are shown at branch nodes. Scale bar: 0.05 substitutions per nucleotide position.

### Functional baseline of the extracellular amylolytic activity

Extracellular amylolytic activity of *Croceibacter* sp. 23-6 was evaluated using a partially purified extracellular amylolytic preparation from the culture supernatant. Activity varied substantially with reaction temperature and pH. Relative activity increased from 30 °C to 50 °C and reached its maximum at 50 °C, followed by a marked decline at higher temperatures (Fig. 3A). The pH profile showed maximum activity at pH 7.0, with lower activity under acidic conditions and reduced activity under alkaline conditions (Fig. 3B).

**Figure 3:**
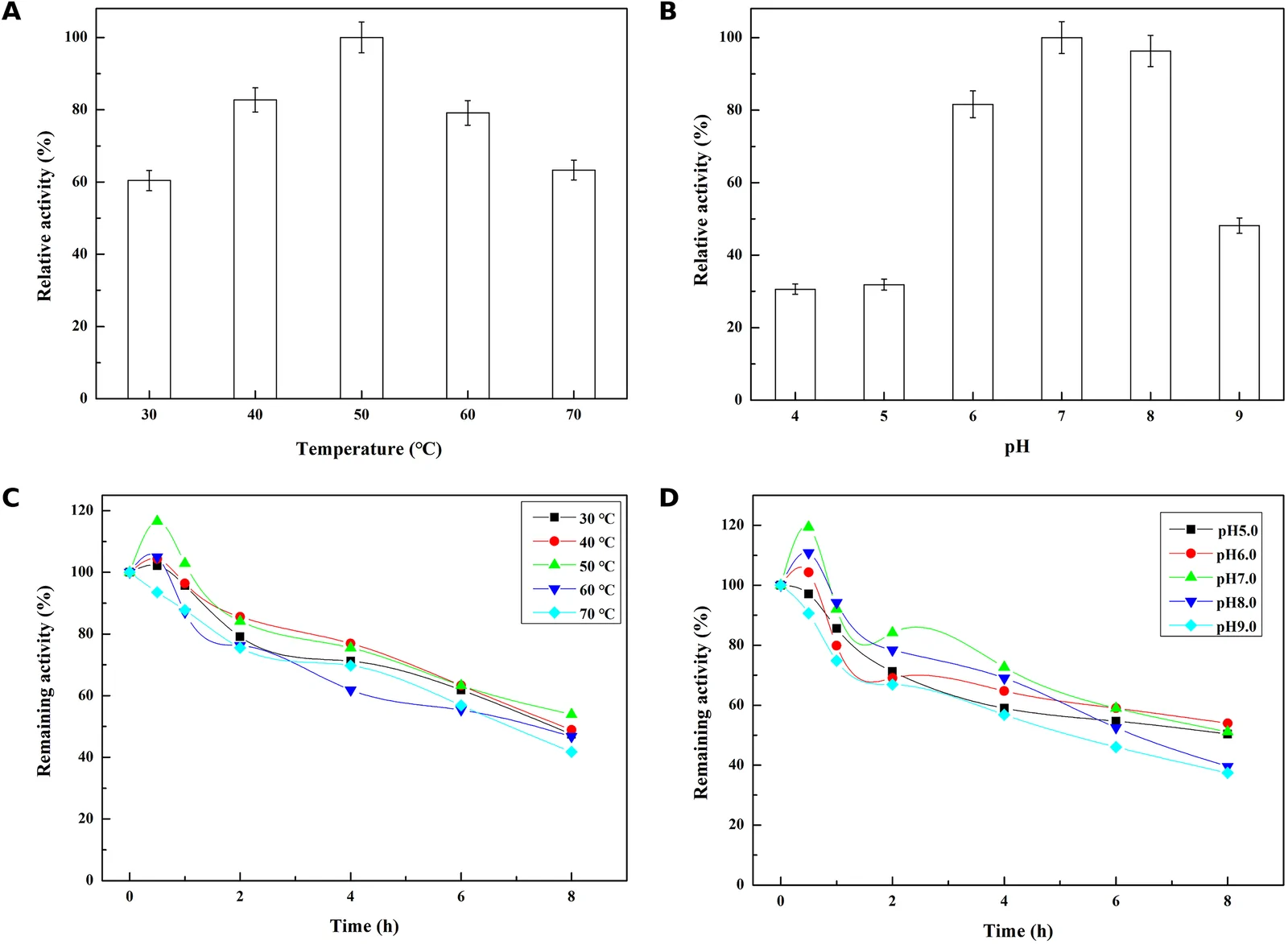
Effects of temperature and pH on activity and stability of the partially purified extracellular amylolytic preparation from *Croceibacter* sp. 23-6. (A) Optimal temperature. (B) Optimal pH. (C) Thermal stability during incubation at different temperatures. (D) pH stability during incubation at different pH values. Relative activity in (A) and (B) is expressed as a percentage of the maximum activity. Residual activity in (C) and (D) is expressed as a percentage of the initial activity. Data are mean ± SD (*n* = 3 independent reaction mixtures).

The amylolytic preparation showed relatively better stability under moderate temperature and near-neutral pH conditions. During 8 h of incubation, the enzyme preparation retained relatively higher residual activity at 50 °C compared with higher temperatures, whereas activity decreased rapidly at 70 °C (Fig. 3C). For pH stability, the enzyme preparation maintained relatively higher residual activity under near-neutral pH conditions, particularly at pH 6.0–7.0, than under more acidic or strongly alkaline conditions (Fig. 3D). These results define the basic functional range of the partially purified extracellular amylolytic preparation under moderate temperature and near-neutral pH conditions.

### Metal-ion effects and apparent kinetic characterization of the partially purified amylolytic preparation

Metal-ion effects were first evaluated at a final concentration of 3 mM. Among the tested ions, Cu^2+^ showed an increased measured activity, reaching 119.0 ± 3.95% of the control value (Fig. 4A). In contrast, Mn^2+^, K^+^, Zn^2+^ and Fe^2+^ reduced the measured activity to 82.37 ± 3.26%, 59.92 ± 2.17%, 51.86 ± 1.86% and 66.75 ± 2.59%, respectively.

**Figure 4:**
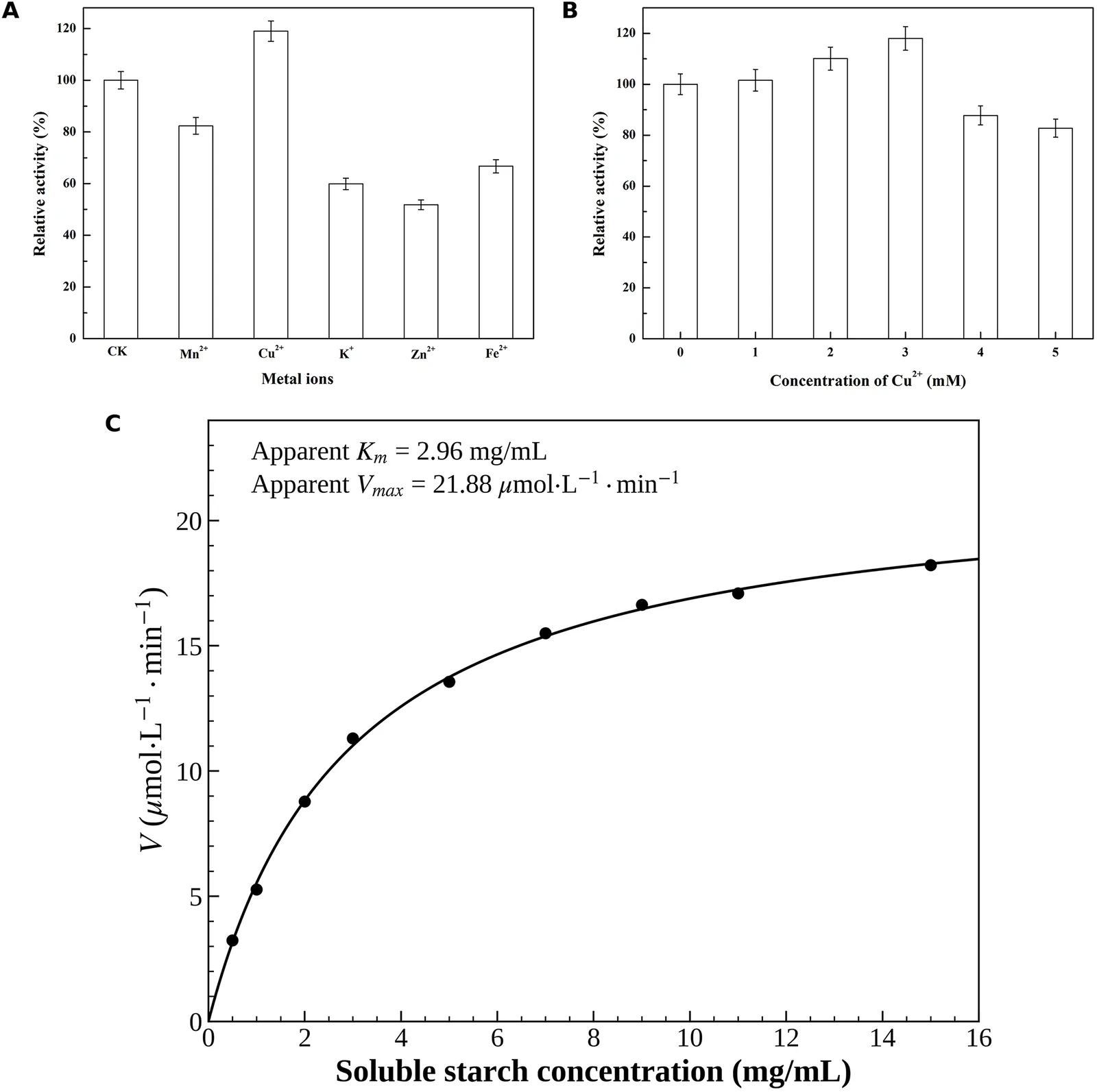
Effects of metal ions and apparent kinetic parameters of the partially purified extracellular amylolytic preparation. (A) Effect of various metal ions at 3 mM on enzyme activity; CK, ion-free control. (B) Effect of different Cu^2+^ concentrations. (C) Michaelis–Menten plot for the hydrolysis of soluble starch. Data points represent the mean ± SD of three independent reaction mixtures. The solid curve represents the nonlinear Michaelis–Menten fit to the replicate-level initial-velocity data. The apparent kinetic parameters (*K_m_* and *V_max_*) are shown in the panel.

Because Cu^2+^ showed the highest measured activity increase in the initial assay, its concentration-dependent effect was further examined. Relative activity increased with Cu^2+^ concentration up to 3 mM, where the highest measured relative activity was observed, and then decreased at higher concentrations (Fig. 4B). By contrast, the measured relative activities in the K^+^, Mn^2+^, Zn^2+^ and Fe^2+^ treatments generally decreased as ion concentration increased (Fig. S1). This pattern indicates a concentration-dependent Cu^2+^-responsive activity profile of the partially purified amylolytic preparation under the tested assay conditions.

Apparent kinetic parameters were then determined using soluble starch as the substrate under the optimum assay conditions. Nonlinear Michaelis–Menten fitting of the replicate-level initial-velocity data yielded an apparent *K*_*m*_ of 2.96 mg/mL (95% CI [2.84–3.08] mg/mL) and an apparent *V*_*max*_ of 21.88 μmol·L^−1^·min^−1^ (95% CI [21.60–22.16] μmol·L^−1^·min^−1^) (Fig. 4C). These kinetic values provide quantitative descriptors of the tested extracellular amylolytic preparation under the specified assay conditions.

### Relative reducing-sugar release from different starch substrates and single-factor evaluation of wheat starch hydrolysis

The relative reducing-sugar release of the partially purified extracellular amylolytic preparation varied markedly among the starch substrates tested (Fig. 5A). With activity toward soluble starch defined as 100%, wheat starch showed the highest relative reducing-sugar release among the insoluble starch substrates, reaching 94.6 ± 4.23% relative reducing-sugar release. Moderate relative reducing-sugar release values were observed for glutinous rice starch, corn starch and potato starch, with relative values of 52.5 ± 2.03%, 47.8 ± 1.99% and 44.9 ± 1.85%, respectively. In contrast, pea starch and cassava starch showed lower relative reducing-sugar release values, at 9.3 ± 0.40% and 9.0 ± 0.37%, respectively. On this basis, wheat starch was selected as the substrate for process-level hydrolysis evaluation.

**Figure 5:**
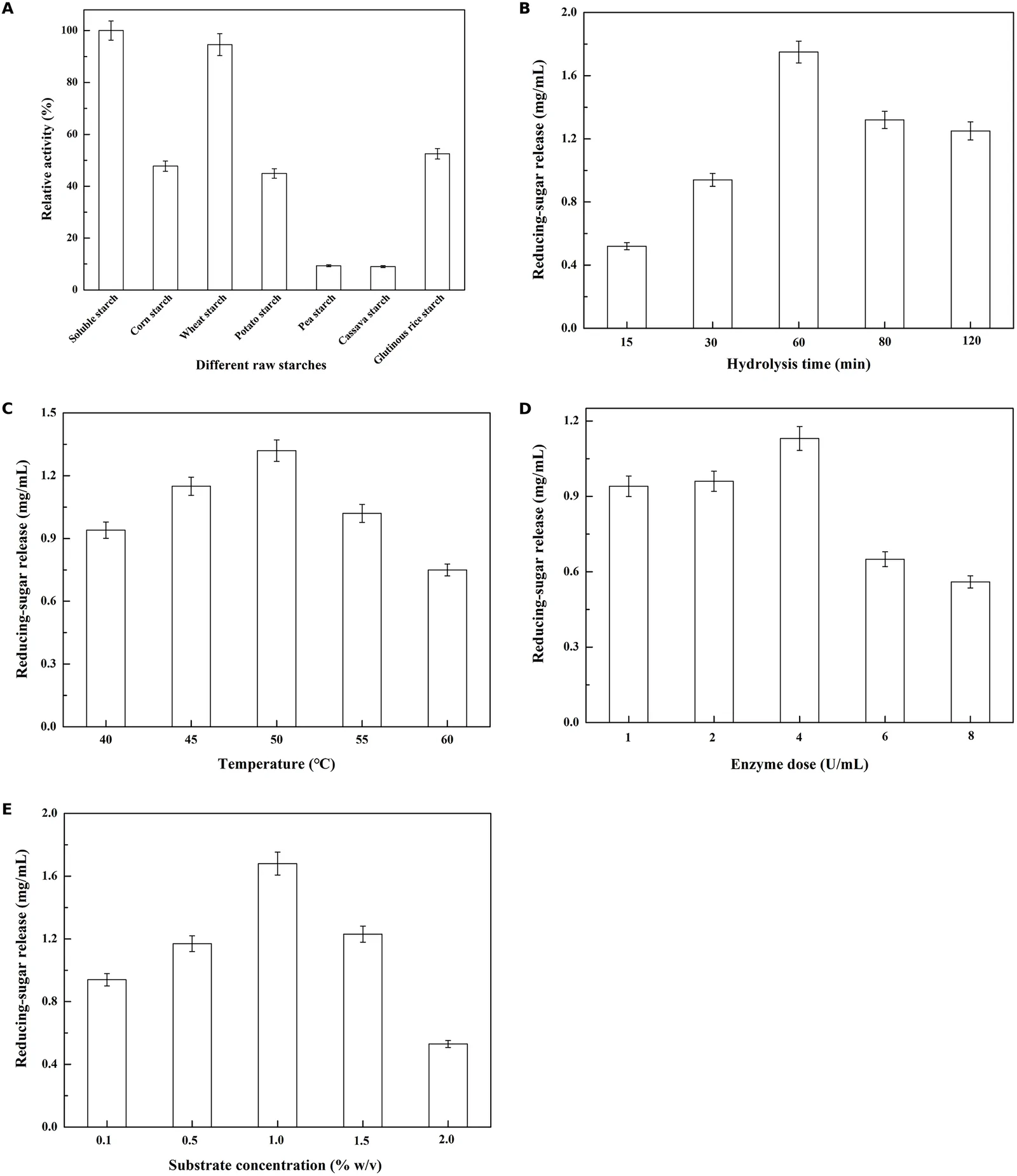
Relative reducing-sugar release from different starch substrates and single-factor analysis of wheat starch hydrolysis by the partially purified extracellular amylolytic preparation. (A) Relative reducing-sugar release from different starch substrates, with reducing-sugar release from soluble starch defined as 100%. (B–E) Effects of (B) hydrolysis time, (C) temperature, (D) enzyme dose, and (E) substrate concentration, on wheat starch hydrolysis. Reducing-sugar release values were expressed as maltose equivalents determined by the DNS assay. Data are presented as mean ± SD from three independent reaction mixtures.

Single-factor experiments were then conducted to define suitable ranges for response surface optimization (Figs. 5B–5E). Reducing-sugar yield expressed as maltose equivalents varied with hydrolysis time, temperature, enzyme dose and substrate concentration. The highest yield in the time-course experiment was 1.75 ± 0.07 mg/mL at 60 min, whereas the temperature experiment showed a maximum yield of 1.32 ± 0.05 mg/mL at 50 °C. An enzyme dose of 4.0 U/mL produced the highest yield in the enzyme-dose experiment, reaching 1.13 ± 0.05 mg/mL. For substrate concentration, 1.0% wheat starch gave the highest yield of 1.68 ± 0.07 mg/mL. These single-factor results were not used as final optimum conditions but served to define the experimental range for multivariable optimization.

### Process-level validation of wheat starch hydrolysis by response surface methodology

A four-factor, three-level Box–Behnken design was used to evaluate the combined effects of hydrolysis time, temperature, enzyme dose and substrate concentration on wheat starch hydrolysis (Table 1). The experimental design comprised 29 randomized runs, including five centre points, and reducing-sugar yield expressed as maltose equivalents was used as the response variable (Table 2). The data were fitted to a quadratic regression model, which was highly significant (*p* < 0.0001). The model showed strong explanatory and predictive performance, with R^2^, adjusted R^2^ and predicted R^2^ values of 0.9738, 0.9477 and 0.8619, respectively. The lack-of-fit test was not significant (*p* = 0.1438), indicating that the model adequately described the experimental data within the tested range.

**Table 1:** Independent variables and levels used in the Box–Behnken design.

Variable	Coded symbol	Symbol	Level (−1)	Level (0)	Level (+1)
Hydrolysis time (min)	A	t	40	60	80
Temperature (°C)	B	T	48	50	52
Enzyme dose (U/mL)	C	[E]	3	4	5
Substrate concentration (% w/v)	D	[S]	0.8	1.0	1.2

**Table 2:** Box–Behnken design matrix and experimental reducing-sugar release expressed as maltose equivalents during wheat starch hydrolysis.

Run order	Coded variables				Reducing-sugar release (maltose equivalents, mg/mL)
	A: t	B: T	C: [E]	D: [S]	
1	0	1	−1	0	1.22 ± 0.05
2	0	0	0	0	2.37 ± 0.08
3	1	0	−1	0	1.44 ± 0.06
4	−1	0	0	1	2.05 ± 0.07
5	0	−1	1	0	1.51 ± 0.06
6	0	−1	0	−1	1.52 ± 0.06
7	−1	−1	0	0	1.77 ± 0.05
8	0	0	0	0	2.46 ± 0.09
9	0	0	0	0	2.36 ± 0.08
10	0	0	0	0	2.41 ± 0.09
11	1	0	0	1	1.66 ± 0.05
12	0	1	0	−1	1.22 ± 0.04
13	−1	1	0	0	1.60 ± 0.05
14	0	0	1	−1	1.78 ± 0.06
15	0	0	−1	1	1.48 ± 0.05
16	0	0	−1	−1	2.03 ± 0.07
17	0	−1	0	1	1.54 ± 0.06
18	0	−1	−1	0	1.43 ± 0.05
19	1	−1	0	0	1.65 ± 0.06
20	−1	0	0	−1	2.07 ± 0.08
21	1	0	1	0	1.53 ± 0.05
22	0	1	0	1	1.02 ± 0.03
23	−1	0	−1	0	1.92 ± 0.07
24	0	1	1	0	1.06 ± 0.03
25	0	0	0	0	2.51 ± 0.09
26	1	0	0	−1	1.69 ± 0.06
27	−1	0	1	0	2.03 ± 0.07
28	1	1	0	0	1.04 ± 0.03
29	0	0	1	1	2.00 ± 0.07

**Note:**

The independent variables are expressed in coded levels (−1, 0, and +1). The corresponding actual values for each level are as follows: hydrolysis time (t), 40, 60, and 80 min; temperature (T), 48 °C, 50 °C, and 52 °C; enzyme dose ([E]), 3, 4, and 5 U/mL; and substrate concentration ([S]), 0.8, 1.0, and 1.2% (w/v). Reducing-sugar release represents the experimentally measured response and is expressed as maltose equivalents. Reducing-sugar release values are presented as mean ± standard deviation (SD) from triplicate determinations.

The final predictive model in terms of coded factors was expressed as follows:

Reducing-sugar yield = 2.42 − 0.2025A − 0.1883B + 0.0325C − 0.0467D − 0.1100AB − 0.0050AC − 0.0025AD − 0.0600BC − 0.0550BD + 0.1925CD − 0.2489A^2^ − 0.7327B^2^ − 0.3764C^2^ − 0.2977D^2^

where A, B, C and D represent hydrolysis time, temperature, enzyme dose and substrate concentration, respectively.

ANOVA showed that the linear terms for hydrolysis time (A) and temperature (B) were highly significant (*p* < 0.0001), and all quadratic terms were significant (Table 3). Among the interaction terms, AB and CD were significant (*p* < 0.05). The perturbation plot indicated that hydrolysis time and temperature exerted stronger effects on reducing-sugar yield than the other variables within the tested range (Fig. 6A). The response surface plots further illustrated the significant interactions between hydrolysis time and temperature and between enzyme dose and substrate concentration (Figs. 6B, 6C).

**Table 3:** Analysis of variance for the quadratic regression model fitted to wheat starch hydrolysis.

Source	Sum of squares	df	Mean square	*F*-value	*p*-value
Model	5.17	14	0.369	37.24	<0.0001
A: t	0.4921	1	0.4921	49.66	<0.0001
B: T	0.4256	1	0.4256	42.96	<0.0001
C: [E]	0.0127	1	0.0127	1.28	0.2771
D: [S]	0.0261	1	0.0261	2.64	0.1267
AB	0.0484	1	0.0484	4.88	0.0443
AC	0.0001	1	0.0001	0.0101	0.9214
AD	0	1	0	0.0025	0.9606
BC	0.0144	1	0.0144	1.45	0.248
BD	0.0121	1	0.0121	1.22	0.2878
CD	0.1482	1	0.1482	14.96	0.0017
A^2^	0.4019	1	0.4019	40.56	<0.0001
B^2^	3.48	1	3.48	351.4	<0.0001
C^2^	0.9191	1	0.9191	92.75	<0.0001
D^2^	0.5747	1	0.5747	58	<0.0001
Residual	0.1387	14	0.0099		
Lack of fit	0.1228	10	0.0123	3.09	0.1438
Pure error	0.0159	4	0.004		
Cor total	5.3	28			

**Note:**

A, B, C, and D represent the coded independent variables for hydrolysis time, temperature, enzyme dose, and substrate concentration, respectively. The statistical significance of model terms was evaluated at *p* < 0.05.

**Figure 6:**
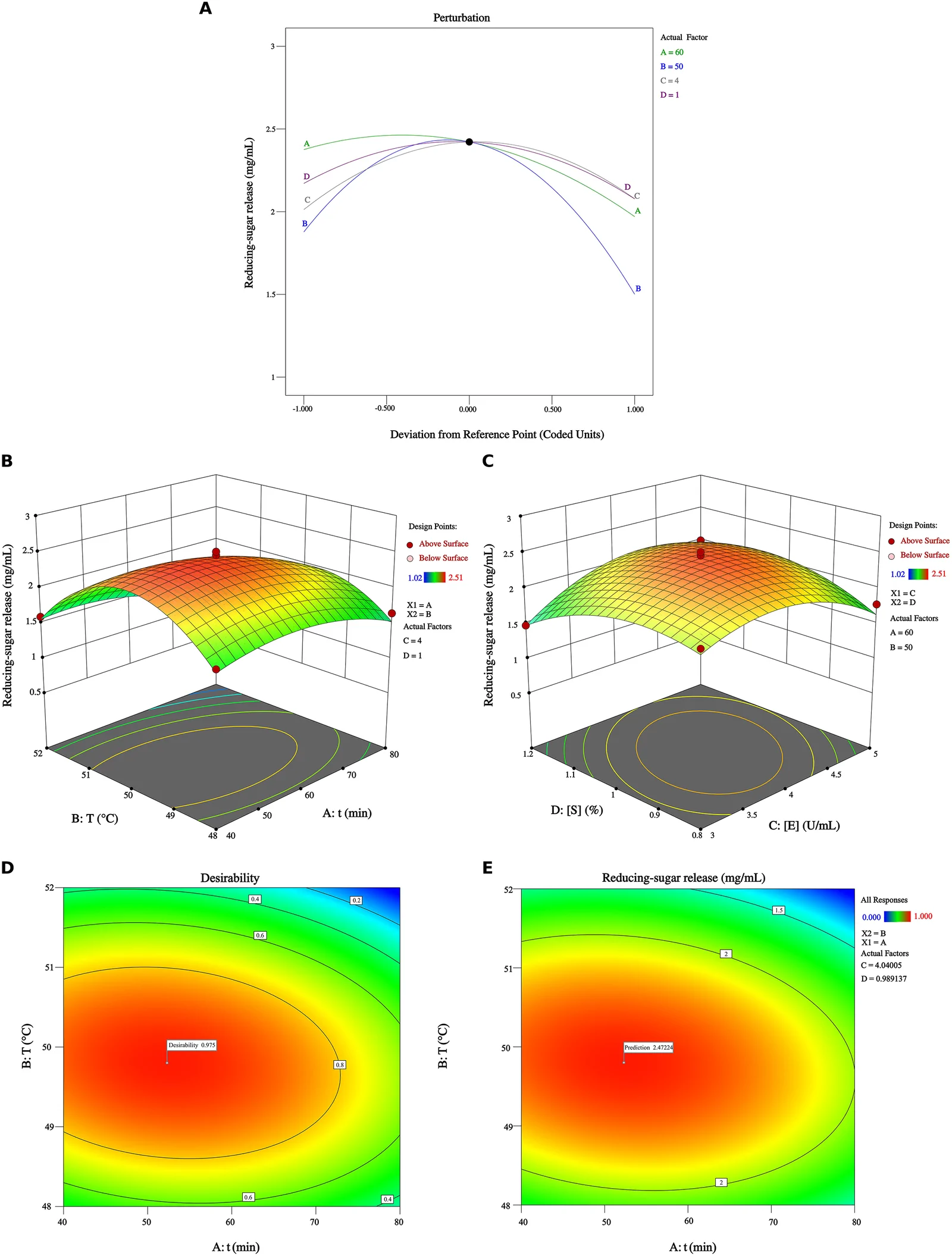
Response surface methodology optimization for wheat starch hydrolysis. (A) Perturbation plot showing the relative influence of coded independent variables (A: hydrolysis time, B: temperature, C: enzyme dose, D: substrate concentration). Steeper slopes for A and B indicate stronger linear effects. (B, C) Three-dimensional response surface plots showing the significant interaction effects between (B) A and B (*p* = 0.0443) and (C) C and D (*p* = 0.0017) on reducing-sugar release. (D) Desirability contour plot for hydrolysis time (A) and temperature (B), showing an overall desirability of 0.975 at the numerical optimum. (E) Numerical optimization solution predicting a maximum reducing-sugar release of 2.47 mg/mL at 52.3 min, 49.8 °C, 4.04 U/mL enzyme and 0.989% (w/v) wheat starch, with an overall desirability of 0.975. Reducing-sugar release values were expressed as maltose equivalents determined by the DNS assay.

Numerical optimization predicted optimum coded conditions of A = −0.385, B = −0.099, C = 0.039 and D = −0.056, corresponding to 52.3 min, 49.8 °C, 4.04 U/mL enzyme and 0.989% wheat starch. The numerical optimum predicted reducing-sugar yield of 2.47 mg/mL, with an overall desirability of 0.975 (Figs. 6D, 6E). For practical validation, the conditions were adjusted to 52 min, 50 °C, 4.0 U/mL enzyme and 1.0% wheat starch. At these practical conditions, the model predicted a reducing-sugar yield of 2.46 mg/mL, with a 95% prediction interval of 2.31–2.62 mg/mL. The validation experiment yielded 2.43 ± 0.09 mg/mL reducing sugars expressed as maltose equivalents (*n* = 3), which fell within the model prediction interval. These results support the applicability of the response surface model for describing wheat starch hydrolysis by the partially purified extracellular amylolytic preparation within the defined experimental range.

## Discussion

This study characterizes *Croceibacter* sp. 23-6 as a culturable marine isolate exhibiting experimentally confirmed extracellular amylolytic activity and evaluates its starch-hydrolysing capacity under defined laboratory conditions. The current study focuses on experimentally supported biochemical characterization rather than establishing industrial applicability of the isolate. Specifically, the work evaluates three aspects: extracellular amylolytic activity of a marine *Croceibacter* isolate, the Cu²^+^-responsive activity profile of a partially purified extracellular amylolytic preparation, and wheat starch hydrolysis under optimized conditions within the tested experimental range.

### Extracellular amylolytic activity in a marine *Croceibacter* isolate

A central contribution of this work is the quantitative biochemical characterization of extracellular amylolytic activity in *Croceibacter* sp. 23-6. Strain 23-6 produced a clear hydrolysis halo on starch agar and measurable amylolytic activity was detected in the cell-free culture supernatant. Together, these observations document extracellular starch-hydrolysing activity in strain 23-6 under the tested conditions. Rather than representing the first phenotypic evidence of starch degradation in the genus, the present study extends previous phenotype-level observations by providing quantitative biochemical characterization of a partially purified extracellular amylolytic preparation from strain 23-6.

This finding expands the experimentally characterized extracellular hydrolytic diversity of a less-studied Flavobacteriaceae lineage. Although members of this family are frequently associated with marine polysaccharide degradation, experimentally validated extracellular hydrolytic activities remain unevenly documented across genera and strains (Thomas et al., 2021; Yang et al., 2025). This unevenness is partly related to differences in polysaccharide-utilization strategies, including extracellular enzyme secretion, cell-associated hydrolysis and substrate-specific uptake systems, which can vary substantially among marine polysaccharide-degrading bacteria (McKee et al., 2021; D’Souza et al., 2023). For *Croceibacter*, phenotypic starch degradation was previously reported for *C. atlanticus* HTCC2559^T^ (Cho & Giovannoni, 2003), and its genome sequence later provided additional metabolic context (Oh et al., 2010).

The present study extends this earlier knowledge by quantitatively characterizing extracellular amylolytic activity and associated biochemical properties in strain 23-6. This strain-level evidence is also consistent with the broader value of culturable environmental microorganisms in enzyme research. Therefore, *Croceibacter* sp. 23-6 represents a culturable marine isolate whose extracellular amylolytic phenotype has been experimentally documented and evaluated under controlled substrate-conversion conditions.

The taxonomic interpretation of strain 23-6 should remain conservative. Its 16S rRNA gene sequence showed 98.95% similarity to *C. atlanticus* HTCC2559^T^, supporting assignment to the genus *Croceibacter* but not definitive species-level identification. Genome-based comparisons would be required to resolve its species status. Accordingly, the taxonomic conclusion of this study is limited to genus-level assignment, whereas the main contribution is functional: it provides direct evidence of extracellular amylolytic activity in *Croceibacter* sp. 23-6 and connects this phenotype to defined biochemical and hydrolysis properties.

### Cu^2+^-responsive activity as the notable biochemical characteristic

The partially purified extracellular amylolytic preparation from *Croceibacter* sp. 23-6 showed maximal activity at 50 °C and pH 7.0, with higher stability under near-neutral to slightly alkaline conditions. These values indicate a moderate catalytic profile rather than an extremophilic one. Therefore, the main biochemical interest of the strain 23-6 preparation lies in its metal-ion response under the tested assay conditions.

The clearest positive metal-ion response was observed with Cu^2+^. At 3 mM, the measured relative activity with Cu^2+^ was 119.0% of the control, whereas K^+^, Mn^2+^, Zn^2+^ and Fe^2+^ were associated with lower measured relative activities. The concentration-dependent assay further showed that the measured activity increased up to 3 mM Cu^2+^ and declined at higher concentrations. This pattern indicates a Cu^2+^-responsive activity profile under the tested assay conditions rather than direct evidence of enzyme activation. Because Cu^2+^-specific analytical-interference controls were not included in the original experiments, the higher DNS-based response observed with Cu^2+^ cannot be attributed unequivocally to enhanced catalytic activity.

This response is notable because Cu^2+^ is commonly reported as inhibitory for α-amylases, although metal-ion effects vary among enzymes. Similar metal-dependent variation has been reported for α-amylase systems, including inhibitory effects of Cu^2+^, Zn^2+^ and Fe^2+^ in barley seed α-amylase assays, further supporting that metal-ion responses are enzyme- and source-dependent rather than uniform across α-amylases (Hossain et al., 2025). Mechanistic and biochemical discussions of Cu²^+^-mediated α-amylase inhibition have generally attributed such effects to interactions with catalytic or structural residues or to metal-induced conformational changes (Pasricha & Gahlot, 2022). As summarized in Table 4, selected microbial and marine-derived α-amylases show divergent Cu^2+^ responses: Cu^2+^ inhibits some bacterial or cold-active marine amylases, whereas Cu^2+^-responsive activity has also been reported in marine actinomycete α-amylase systems (Al-Agamy et al., 2021; Kim et al., 2024; Rajesh & Gummadi, 2024; Bendtsen et al., 2025). The response observed for the 23-6 preparation is therefore best interpreted as a measured Cu^2+^-responsive phenotype under the present assay conditions, not as evidence of a defined molecular mechanism underlying the Cu^2+^ response.

**Table 4:** Comparative characteristics of selected microbial amylolytic systems with emphasis on Cu^2+^ response and starch hydrolysis.

Source/enzyme	Source context	Optimum T/pH	Cu^2+^/metal-ion response	Starch-related endpoint	Reference
*Croceibacter* sp. 23-6 amylolytic preparation	Mesopelagic marine bacterium; extracellular amylolytic activity	50 °C; pH 7.0	Cu^2+^-responsive activity to 119.0% at 3 mM; K^+^, Mn^2+^, Zn^2+^ and Fe^2+^ showed lower measured relative activities	Highest relative reducing-sugar release among tested starch substrates; RSM-validated reducing-sugar release (expressed as maltose equivalents) of 2.43 ± 0.09 mg/mL	Present study
*Streptomyces rochei* α-amylase	Marine actinomycete	65 °C; pH 6.0	Cu^2+^, Zn^2+^ and Fe^2+^ enhanced activity	α-Amylase production and biochemical characterization	Al-Agamy et al. (2021)
Chukchi Sea cold-active amylase	Marine cold-active bacterium	25 °C; pH 7.0	Cu^2+^ and Zn^2+^ inhibited activity; Mn^2+^, Ba^2+^ and Ca^2+^ showed positive effects	Cold-active amylase characterization	Kim et al. (2024)
*Bacillus* sp. PM06 α-amylase	Bacterial α-amylase	50 °C; pH 5.5	Cu^2+^ inhibited activity	Wheat bran saccharification	Rajesh & Gummadi (2024)
*Anoxybacillus karvacharensis* K1 α-amylase	Thermophilic bacterium; recombinant α-amylase	55 °C; pH 6.5	Cu^2+^-selective activation was not the defining feature	High activity toward wheat starch and other starch substrates	Ghevondyan et al. (2024)
*Jeotgalibacillus malaysiensis* D5^T^ AmyJM	Marine-associated bacterial lineage; recombinant GH13_5 α-amylase	40 °C; pH 7.5	CaCl_2_ enhanced activity and stability	Efficient hydrolysis of raw and gelatinized starches, including raw wheat starch	Radzlin et al. (2024)
*Bacillus amyloliquefaciens* α-amylase	Bacterial α-amylase evaluated by Box–Behnken/RSM	NR	Cu^2+^ response was not the main focus	RSM-based optimization of starch hydrolysis	Abd-Elhalim et al. (2023)

**Note:**

NR, not reported or not a central focus of the cited study. Selected studies were compared in terms of source context, optimum conditions, metal-ion response and starch-related performance. Direct quantitative comparison is limited by differences in enzyme preparation, assay conditions, substrates and activity units among studies.

The mechanism underlying Cu^2+^-responsive activity remains unresolved. The decline in measured activity above 3 mM indicates that the response is concentration dependent, but the present assays do not identify the molecular basis of this pattern. Because sequence-level identification, purification to homogeneity, metal-binding residues and structural information are not yet available, the present work should be viewed as identifying a Cu^2+^-responsive activity profile rather than resolving its molecular basis.

### Wheat starch hydrolysis as laboratory-scale validation

The substrate-dependent hydrolysis results linked the extracellular amylolytic phenotype to a defined starch-conversion context. Among the insoluble starch substrates tested, wheat starch showed the highest relative reducing-sugar release, reaching 94.6%, whereas glutinous rice, corn and potato starches showed moderate hydrolysis and pea and cassava starches showed much lower relative reducing-sugar release. This observed hydrolysis response toward wheat starch provided a basis for selecting wheat starch for subsequent optimization rather than treating the response surface experiment as an isolated statistical exercise.

The observed substrate dependence may reflect differences in granule architecture, amylose-to-amylopectin ratio, crystallinity, surface accessibility and exposure of α-1,4-glycosidic linkages. Similar substrate-dependent hydrolysis patterns have been reported for α-amylases showing strong activity toward wheat starch or raw wheat starch (Ghevondyan et al., 2024; Radzlin et al., 2024). Substrate-dependent hydrolysis patterns have also been observed in recent biochemical studies of non-microbial α-amylases, indicating that differences in substrate conversion efficiency should be interpreted in relation to enzyme source and assay conditions (Albalawi et al., 2025). However, the present study did not directly analyse starch granule morphology, gelatinization state, crystallinity or hydrolysis product profiles. Therefore, the higher hydrolysis response observed for wheat starch should be interpreted as an experimentally observed hydrolytic phenotype under the tested conditions, rather than as evidence of a resolved structure–substrate relationship.

The response surface analysis strengthened the study by connecting enzyme phenotype with measurable product formation. The quadratic model was significant, the lack-of-fit was not significant, and the predicted and experimental values were closely aligned. Under the validated conditions of 52 min, 50 °C, 4.0 U/mL enzyme and 1.0% wheat starch, reducing-sugar production expressed as maltose equivalents reached 2.43 ± 0.09 mg/mL, consistent with the model-predicted value of 2.46 mg/mL at the practical validation conditions and within the corresponding 95% prediction interval of 2.31–2.62 mg/mL. The agreement between predicted and experimental values supports the applicability of the response surface model for describing wheat starch hydrolysis under the tested conditions.

Table 4 places this result alongside selected α-amylase studies relevant to wheat starch activity and RSM-based starch hydrolysis optimization. Recent work on recombinant, marine-associated or marine bacterial α-amylases has shown that wheat starch can be a favourable substrate or that raw-starch hydrolysis can be achieved under defined enzymatic conditions (Puspasari et al., 2013; Ghevondyan et al., 2024; Radzlin et al., 2024). Similar RSM-based optimization has been used to improve amylase production from marine or halophilic *Bacillus* strains, supporting the relevance of multivariable optimization for amylase-related process evaluation (Bandal et al., 2021; Abd-Elhalim et al., 2023; Adetiloye et al., 2025). In this manuscript, the RSM component is used as a laboratory-scale functional validation step: strain screening established extracellular starch hydrolysis, biochemical characterization defined the operating range and Cu^2+^ responsiveness, substrate testing identified wheat starch for subsequent optimization based on its highest measured response among the six commercial starch preparations, and multivariable optimization defined and experimentally validated suitable hydrolysis conditions.

### Functional positioning and study limitations

The selected studies summarized in Table 4 provide a concise literature context for interpreting the 23-6 enzyme preparation in terms of source background, optimum profile, metal-ion response and starch-conversion endpoint. This comparison is not intended to rank enzymes quantitatively, because enzyme preparation, assay conditions, substrates and activity units differ among reports. Instead, it clarifies where the 23-6 preparation is functionally distinctive. Recombinant or sequence-characterized enzymes, such as the α-amylase from *Anoxybacillus karvacharensis* K1 and AmyJM from *Jeotgalibacillus malaysiensis* D5^T^, provide stronger molecular-level evidence and detailed substrate-performance information (Ghevondyan et al., 2024; Radzlin et al., 2024). By contrast, the present work is best positioned as a strain-level functional study of a culturable marine *Croceibacter* isolate. Its main contribution is the combined demonstration of extracellular amylolytic activity, Cu^2+^ responsiveness and validated wheat starch hydrolysis.

Several limitations remain. Species-level taxonomic assignment requires genome-based analysis. The preparation was only partially purified, and the responsible enzyme(s) have not been identified. The mechanism of Cu^2+^-responsive activity cannot be inferred from metal-ion assays alone. The wheat starch hydrolysis experiments quantified maltose equivalents but did not resolve the full product spectrum or structural changes in the substrate. In addition, the extracellular preparation used for biochemical characterization was obtained after a fixed 96-h cultivation period; a production time course was not performed, so changes in extracellular amylolytic activity during cultivation were not assessed. These limitations define the boundary between the current phenotypic and laboratory-scale evidence and the molecular questions that remain open.

Future work should focus on sequence-level identification of the responsible amylolytic enzyme(s), purification to homogeneity, heterologous expression, and structural or mutational analysis of potential metal-binding residues. Product analysis by HPLC or related methods would clarify the distribution of glucose, maltose and higher maltooligosaccharides. Evaluation of raw starch digestion, longer-term enzyme stability and compatibility with relevant reaction components would further clarify the practical scope of this enzyme preparation. Overall, the 23-6 enzyme preparation is not positioned as a complete molecularly resolved amylolytic enzyme system, but as a partially purified extracellular amylolytic preparation from a marine *Croceibacter* isolate with experimentally documented Cu^2+^-responsive activity and optimized wheat starch hydrolysis.

## Conclusions

This study provides strain-level evidence that *Croceibacter* sp. 23-6 is a culturable mesopelagic marine isolate with experimentally confirmed extracellular amylolytic activity. The partially purified extracellular amylolytic preparation showed a moderate catalytic profile, with optimal activity at 50 °C and pH 7.0. Among the tested metal-ion conditions, 3 mM Cu^2+^ showed the highest measured relative activity (119.0% of the ion-free control), whereas K^+^, Mn^2+^, Zn^2+^ and Fe^2+^ showed lower measured relative activities. Wheat starch showed the highest relative reducing-sugar release among the starch substrates evaluated relative to soluble starch as the reference, and response surface optimization validated reducing-sugar production from wheat starch, yielding 2.43 ± 0.09 mg/mL expressed as maltose equivalents under the practical validation conditions. These findings connect marine isolate screening, extracellular enzyme characterization, Cu^2+^-responsive activity and wheat starch hydrolysis into a coherent functional evidence chain under defined laboratory conditions. Overall, this work defines an experimentally documented extracellular amylolytic phenotype in *Croceibacter* sp. 23-6 and provides a basis for future sequence-level identification and mechanistic analysis of the responsible amylolytic enzyme(s), rather than establishing a complete molecularly resolved enzyme system.

## Supplemental Information

10.7717/peerj.21854/supp-1 Supplemental Information 1 Source data for enzyme characterization and wheat starch hydrolysis. summarized source data used to generate Figures 3–6 and Supplemental Figure S1. The data include enzyme activity profiles, stability assays, metal-ion response assays, substrate hydrolysis profiles, single-factor wheat starch hydrolysis experiments, Box–Behnken design response values, and ANOVA results for response surface methodology. Mean and standard deviation values are provided where applicable.

10.7717/peerj.21854/supp-2 Supplemental Information 2 Effects of different concentrations of K^+^, Mn^2+^, Zn^2+^ and Fe^2+^ on the activity of the partially purified extracellular amylolytic preparation from *Croceibacter* sp. 23-6. (A) K^+^. (B) Mn^2+^. (C) Zn^2+^. (D) Fe^2+^. Relative activity was calculated relative to the ion-free control, defined as 100%. Data are presented as mean ± SD from three independent reaction mixtures.

## Additional Information and Declarations

### Data Availability

The following information was supplied regarding data availability:

The data is available in the Supplemental Files.

The 16S rRNA gene sequence of *Croceibacter* sp. 23-6 is available in GenBank under accession number: PX796489.
